# Associations between Family Adversity and Brain Volume in Adolescence: Manual vs. Automated Brain Segmentation Yields Different Results

**DOI:** 10.3389/fnins.2016.00398

**Published:** 2016-09-05

**Authors:** Hannah Lyden, Sarah I. Gimbel, Larissa Del Piero, A. Bryna Tsai, Matthew E. Sachs, Jonas T. Kaplan, Gayla Margolin, Darby Saxbe

**Affiliations:** ^1^Department of Psychology, University of Southern CaliforniaLos Angeles, CA, USA; ^2^Department of Psychology, Brain and Creativity Institute, University of Southern CaliforniaLos Angeles, CA, USA

**Keywords:** amygdala, hippocampus, methodology, family aggression, early life stress, adolescence

## Abstract

Associations between brain structure and early adversity have been inconsistent in the literature. These inconsistencies may be partially due to methodological differences. Different methods of brain segmentation may produce different results, obscuring the relationship between early adversity and brain volume. Moreover, adolescence is a time of significant brain growth and certain brain areas have distinct rates of development, which may compromise the accuracy of automated segmentation approaches. In the current study, 23 adolescents participated in two waves of a longitudinal study. Family aggression was measured when the youths were 12 years old, and structural scans were acquired an average of 4 years later. Bilateral amygdalae and hippocampi were segmented using three different methods (manual tracing, FSL, and NeuroQuant). The segmentation estimates were compared, and linear regressions were run to assess the relationship between early family aggression exposure and all three volume segmentation estimates. Manual tracing results showed a positive relationship between family aggression and right amygdala volume, whereas FSL segmentation showed negative relationships between family aggression and both the left and right hippocampi. However, results indicate poor overlap between methods, and different associations were found between early family aggression exposure and brain volume depending on the segmentation method used.

## Introduction

Early adversity is known to compromise mental and physical health across the lifespan (Felitti et al., 1998). Moreover, children exposed to “risky family” environments show deficits in emotion regulation, social competence, and dysregulated stress responding (Repetti et al., 2002). Over the last several decades, researchers have explored many potential mechanisms of the effects of risky family environments. Altered brain structure and function due to early life stress is one possible mechanism.

The hippocampus is a brain region critically important to memory (Markowitsch and Pritzel, 1985) and is known to be modulated by stress or adversity exposure, possibly through the influence of stress hormones. Smaller hippocampi have been found in adults who have experienced early adversity (Wolkowitz et al., 1990; Newcomer et al., 1994; Keenan et al., 1996; Bremner et al., 1997; Perry, 2001; Heim et al., 2002; Lupien et al., 2005; Teicher et al., 2006), but not in children (Carrion et al., 2001; De Bellis, 2001; De Bellis et al., 2002; Woon and Hedges, 2008), nor in adolescents (Carrion et al., 2001; De Bellis, 2001; De Bellis et al., 2002; Woon and Hedges, 2008; Frodl et al., 2010; Rao et al., 2010).

The amygdala is a subcortical limbic system structure that has been implicated in learning about the salience and emotional significance of stimuli (Davis and Whalen, 2001). Similarly to the hippocampus, the amygdala has a protracted development extending into late childhood and undergoes a refinement of activation during childhood exhibited by higher activation of the amygdala to neutral (vs. emotional) faces in children than adults (Thomas et al., 2001; Lobaugh et al., 2006). Lesion studies have found that early damage to the amygdala leads to deficiencies in fear learning later in life, as well as significant impairment in processing of facial expression (Adolphs et al., 1994), whereas this has not been found with later occurring insults (Adolphs et al., 1994; Hamann et al., 2002). Smaller amygdalae have been found in adults who experienced childhood adversity (Driessen et al., 2000; Schmahl et al., 2003). Larger amygdalae have been found in populations of children affected by aversive caregiving (Mehta et al., 2009; Tottenham et al., 2009a,b). These results are consistent with animal research and human studies that have found larger amygdala volume and activity due to early life stress in children (Vyas et al., 2004; Yang et al., 2007; Tottenham and Sheridan, 2010). The research suggests that the amygdala undergoes expansive growth and hyperactivity after a stressor, which may lead to larger volume measured by MRI in childhood. Subsequently, after a prolonged period this hyperactivity and increase in glucocorticoid response may result in cellular atrophy and smaller volumes measured by MRI in adulthood. The timing of measurement (i.e., in childhood, adolescence, or adulthood), may play a role in how different methodologies quantify different brain structures, given the differences in brain size, shape, or a differing pace of growth of different structures during adolescence (Casey et al., 2008).

Both the hippocampus and the amygdala have been extensively studied in regards to potential volume differences associated with early adversity. However, results of these studies have been mixed. The relationship between childhood adversity and brain volume in adolescence remains unclear. Additionally, studies have typically looked at severe adversity (such as child abuse or institutional care), and the potential effects of mild-to-moderate family aggression on brain structure have not been explored. The current study aimed to investigate the relationship between early life aggression exposure and brain volume in adolescence.

Not only have previous studies on early adversity and the brain reported varying results, but they also differ in important methodological ways. These differences may be due to: (1) the point in development at which the MRI was acquired, i.e., during adolescence or during adulthood, and (2) varying brain volume measurement techniques. The fact that different methodologies for brain segmentation lead to different results introduces a major limitation in the structural MRI literature. No previous studies have compared brain segmentation approaches in a sample of adolescents, a time of dynamic brain growth.

### Approaches used to estimate brain volume

Differences in segmentation method may play a role in the conflicting results that have emerged within the literature on early life aggression exposure and brain volume. While automated segmentation uses previously determined probabilistic maps of brain structures to segment and label study-specific brains, manual tracing involves hand-tracing regions of interest (ROI) based on the individuals' brain structure using predetermined landmarks and has been considered the “gold standard” for brain structure research. However, because manual tracing is time-intensive, several software packages have been developed to facilitate anatomical analysis of brain structure. The Oxford Centre for Functional MRI of the Brain (FMRIB) Software Library (FSL) is a set of tools that includes software for automated segmentation to give volumetric output of structures based on already defined atlases. Similarly, FreeSurfer algorithms perform semi-automated subcortical and cortical segmentation and assign a neuroanatomical label to each voxel based on probabilistic information automatically estimated from a large training set of expert measurements (Fischl et al., 2002). NeuroQuant is a relatively new software package that uses the same algorithms as FreeSurfer, but uses an age-matched atlas that allows for greater variability in anatomy due to brain development, age, or gender, and unlike FreeSurfer is a fully automated process (Kovacevic et al., 2009). Based on comparisons to expert manual segmentation, NeuroQuant is also FDA approved for clinic use.

Studies have compared automated segmentation and manual tracing of brain structures (Heckemann et al., 2006; Jatzko et al., 2006; Han and Fischl, 2007; Barnes et al., 2008; Powell et al., 2008; Aljabar et al., 2009; Artaechevarria et al., 2009; Fellhauer et al., 2015). These comparison studies use manual segmentation as a reference and validate the automated techniques based on the manually segmented volumes. Comparisons are made based on the spatial overlap, volume similarity, as well as correlations between methods. Although no standard metric for comparing segmentation methods has yet to be established, overlap measurements range from about 70 to 90% (Morey et al., 2009; Dewey et al., 2010; Sánchez-Benavides et al., 2010; Cabezas et al., 2011), while correlations between methods range from 0.76 to 0.90 (Barnes et al., 2008; Dewey et al., 2010; Sánchez-Benavides et al., 2010; Shen et al., 2010; Doring et al., 2011; Mulder et al., 2014), for structures including hippocampus, amygdala, putamen, and ventricles.

Although many studies have compared the overlap and correlation between methods, only two studies have compared the associations between a variable of interest and brain volume using different methods and both found different results depending on the method chosen (Dewey et al., 2010; Mulder et al., 2014). Dewey et al. (2010) concluded that although the methods showed overlap within the range that has previously been found, different methods led to different correlations with clinical measures of interest. Similarly, Morey et al. (2009) compared differences in hippocampal volume in patients with major depressive disorder vs. controls with two different automated methods after they were shown to have significant overlap with manual segmentations. Smaller hippocampal volumes were found in individuals with depression using FreeSurfer, but not FSL. Notably, the comparison between methods would suggest that either method could be used, but the findings of the analysis differed depending on which method was chosen. These results suggest that: (1) there exist differences in volume estimation given different methods of segmentation and (2) even if these differences are “close enough” to one another to use automated vs. manual segmentation, relationships with clinical covariates might be different given the different methods used.

Few comparison studies have focused solely on adolescents. Although automated segmentation has been shown to be comparable to manual tracing in adult populations, (e.g., Fischl et al., 2002; Morey et al., 2009; Seixas et al., 2010) it is less well documented whether automated segmentation works in adolescents given that they may have differences in brain size, shape, or a differing pace of growth of different structures (Casey et al., 2008). Given that adolescent brains are smaller than adult brains, and that subcortical structures may be developing ahead of cortical ones, it is imperative to compare methodologies in adolescent as well as adult samples.

Given the lack of research on the relationship between family aggression exposure in early life and brain volume in adolescence, as well as the possibility that the results found may depend on the choice of method, the current study aimed to address whether: (1) different methodologies used for segmentation would overlap in a sample of adolescents and (2) if these different methods would lead to similar or disparate results when exploring the association between family aggression exposure in early life and brain volume in adolescence.

The current study focused on a sample of adolescents who were part of a larger longitudinal study. Rating of family aggression (both marital and parent-child aggression) were collected from both parents and youth, when the youth were 12–13 years old. The youth were scanned an average of 4 years later. Hippocampi and amygdalae were segmented using three different techniques, manual tracing, FSL (v.5.0.8), and NeuroQuant (v1.4). These two automated methods were selected because FSL is one of the most widely available software, and NeuroQuant represented an entirely automated program with a personalized segmentation procedure. FreeSurfer is another commonly used segmentation program that uses the same algorithm for segmentation as NeuroQuant. NeuroQuant was chosen for this particular study given its FDA approval as well as its use of an age-matched atlas. Linear regression analyses were run to assess the relationship between early life aggression exposure and hippocampal and amygdalae volume utilizing the three different methods. We hypothesized the following:

H1: Consistent with the previous literature, we expect family aggression to be associated with smaller hippocampal volumes and larger amygdalae volumes measured in adolescence, although this may differ by method.
H2: Consistent with past research on adults, we expect that brain volumes estimated by manual tracing, FSL, and NeuroQuant will be in general agreement. However, given that adolescent brains are typically smaller and that different structures develop at different rates, we expect more discrepancies between the three methods than have been found in comparative studies using adult samples. Similarly, we expect that if discrepancies exist in the segmentation estimates, these discrepancies may lead to different relationships between early life aggression exposure and hippocampal and amygdalae volume.

## Methods

### Participants

Participants were recruited as part of a second cohort from a longitudinal study of family environments and youth development (Margolin et al., 2010). Recruited primarily through newspaper ads and word of mouth, families needed to meet the following criteria to be eligible: Both parents and a child entering or in middle school needed to live together for the past 3 years and all three family members had to be able to complete questionnaires in English. Participants for the current study were recruited from a subset of 43 families who participated in a family discussion in their second wave of study participation (mean age = 15.51 years). The eligibility criteria for the MRI portion of the study included that youth not have metal in their body that would be contraindicated for MRI scanning, and not be currently taking any psychoactive medications. Of the 43 families contacted, seven youth were ineligible, five declined to participate, and seven could not be reached or scheduled. Twenty-four youth participated in the scanning study, but one youth did not have useable structural data because of poor image quality. Thus, the final sample consisted of 23 adolescents. One was left-handed, 14 (58%) were male, and they averaged 17.05 years of age (range 15.47–18.72). All analyses were run with and without the left-handed individual and results were the same, thus he was kept in the final sample. The sample was diverse, reflective of the urban community from which it was drawn: 9 youth (39.1%) identified as Caucasian, 5 youth (21.7%) identified as Hispanic/Latino, 5 youth (21.7%) identified as multiracial, 3 youth (7.6%) identified as African-American, and 1 youth (0.04%) identified as Asian American. The participants were from relatively affluent backgrounds on average, although there was a wide range of parental incomes: (mean income = $110,014; *SD* = $71,738; range = $8000–$255,000). These income data are in line with the large urban recruitment area, where the cost of living ranks 36.4% above the national average (U. S. Census Bureau, 2010) and median household income for families is $62,595, with 29.3% of families reporting incomes above $100,000 (U. S. Census Bureau, 2011). Socioeconomic status (SES) was included as a covariate in all analyses and did not explain any additional variance; thus it was not included in the final analyses.

### Procedure

Family aggression data were collected using a multi-rater, multi-domain approach during the first visit of the longitudinal study when the youth were on average, 12.87 years old (*SD* = 0.70). Both parents and the youth came to the lab for a 3–4 h visit, and completed questionnaires among other procedures. Approximately four years later (mean = 4.0 years, *SD* = 0.45 years, range = 3.2–5 years), the subset of youth returned for the MRI scan. All participants were scanned for ~2 h using a battery that included functional, resting state, and structural scans.

### Scanner protocol

Whole brain images were acquired using a Siemens 3 Tesla MAGNETON TIM Trio Scanner with a 12-channel matrix head coil. Anatomical images were acquired using a magnetization prepared rapid acquisition gradient (MPRAGE) sequence (TI = 900 ms, TR = 1950 ms, TE = 2.26 ms, flip angle = 7°), isotropic voxel resolution of 1mm.

### Measures

#### Family aggression exposure

A composite family aggression variable was created using multi-rater reports of both mother-father conflict and parent-child conflict. Specifically, we combined assessments of frequency of aggressive behaviors between the spouses [from the Domestic Conflict Index (DCI); Margolin et al., 1998] and between parents and children within the previous year [adapted from the Conflict Tactics Scale—Parent/Child (PCC); Straus et al., 1998]. Both parents and the youth reported on parents' spousal aggression. Parents reported on their own behavior and their partners' behavior, and youth reported on the behavior of each parent. The spousal aggression questionnaire asked how many times, over the previous year, 42 different aggressive behaviors had occurred. These items included 17 physical aggression items (e.g., shaking or slapping the spouse) and 25 emotional aggression items (e.g., swearing at the spouse).

For the Conflict Tactics Scale—Parent/Child, fathers and children reported on father-child aggression and mothers and children reported on mother-child aggression. This questionnaire asked how many times during the previous year any of 17 aggressive behaviors had occurred; these included 7 physical aggression items (e.g., shaking or slapping the child) and 10 emotional aggression items (e.g., swearing at a child; threatening to kick a child out of the house).

Before computing final scores, a maximum reporter variable was created for mother and father's behavior for each item. On both questionnaires the highest number of incidences that each participant (mother, father, or child) reported for that item was chosen as the endorsement for that item. This strategy has been utilized in other studies of family aggression to help account for underreporting of socially undesirable behavior (Margolin et al., 2010). Parent-parent and parent-child aggression were combined since these two forms of aggression are often highly correlated within families, and our goal was to capture the overall climate of aggression within the family (Margolin and Gordis, 2000; Margolin et al., 2001, 2010). In order to combine the DCI and the PCC, each item was changed to a 0–3 scale based on the maximum item endorsement: 0 = the item never occurred in the past year; 1 = once in the past year; 2 = 2–5 times in the past year; 3 = 6 + times in the past year. This scoring approach was designed to maximize normality and reliability, and has been previously used to document family aggression within the larger longitudinal study from which the study sample was drawn (Margolin et al., 2010). This was done for both questionnaires, and separately for mother's behavior and father's behavior. Means across items were then taken for each questionnaire and each parent: DCI Mother Behavior (mean = 0.52, *SD* = 0.22, range = 0.02–0.90), DCI Father Behavior (mean = 0.41, *SD* = 0.21, range = 0.00–0.90), Conflict Tactics Scale-Parent/Child Mother Behavior (mean = 0.72, *SD* = 0.52, range = 0.0–2), Conflict Tactics Scale-Parent/Child Father Behavior (mean = 0.46, *SD* = 0.50, range = 0.00–1.7). These scores were then averaged to create an aggregate family aggression variable that combined both parents' behavior over both domains (marital and parent-child conflict; mean = 0.52, *SD* = 0.28, range = 0.01–1.11).

### Analyses

#### Preprocessing

For automated procedures the T1 image for each participant was brain extracted using FSL's Brain Extraction Tool (BET). Before any manual or automated segmentation was performed brains were realigned (but not resized: See Allen et al., 2008 for discussion) along a plane running through the anterior and posterior commissures (i.e., the AC–PC line). The anterior commissure (AC), posterior commissure (PC), and center of the brain were found manually and the brain was rotated to put the AC and PC on the same horizontal and vertical plane. This procedure is commonly used in structural brain analysis and ensures that coronal slices in all subjects are perpendicular to a uniformly and anatomically defined axis of the brain (Allen et al., 2008). FSL segmentation and manual segmentation were performed with these AC–PC aligned images. NeuroQuant pipelines necessitate the use of raw DICOM files directly from the scanner in the analysis, and thus these images were not AC–PC aligned. FSL's FAST (FSL's automated segmentation tool) was also used to segment each participant's brain into white matter, gray matter, and CSF. Total brain volume (TBV: Gray matter plus white matter) was calculated using the extracted volumes and ratios were calculated for each extracted segmentation. The ratio of each segmentation to TBV was used in all analyses.

#### Automated segmentation

##### FSL

FSL's FIRST (Patenaude et al., 2011; FMRIB's Integrated Registration and Segmentation Tool) was used to automatically segment T1 images into anatomical ROIs for the amygdala and the hippocampus. The program uses FSL utilities to segment the brain into discrete subcortical structures. After the brain was segmented, command line functions from FSL's software package, specifically fslstats, were used to extract volume data for the amygdala and the hippocampus, as defined by the Harvard-Oxford Subcortical Atlas.

##### NeuroQuant segmentation

NeuroQuant software package (CorTechs Labs, La Jolla, California) is a fully automated, deterministic approach to MRI segmentation. It has received US Food and Drug Administration (FDA) 510K clearance for clinical use to measure MRI volumes of brain structures. The algorithm used in the NeuroQuant software includes: (1) a quality control step that determines whether the MR imaging sequence conforms to the specifications required to perform automated segmentations, (2) a correction for gradient non-linearity and B1 field inhomogeneity, and (3) skull stripping the MRI images followed by automated segmentation of anatomic structures. The segmentation procedure involves automated methods (used by FreeSurfer) that rely on probabilistic atlas-based segmentation. Field maps were not collected for the purposes of the current study, therefore gradient non-linearity and B1 field inhomogeneity correction was not conducted. After the brain was segmented, segmentations were reviewed visually for accuracy and none were rejected. Volumetric output (in mm^3^) was then exported for each bilateral amygdalae and hippocampi segmentation.

#### Manual segmentation

The following anatomical procedures were used to manually trace bilateral amygdalae and hippocampi.

##### Anatomical definition of the hippocampus

The neuroanatomical criteria chosen for hippocampal delineation were taken from existing protocols (Narr et al., 2004). The hippocampi were traced in coronal brain slices from anterior to posterior, using fslview tools. All three (sagittal, coronal, and axial) planes were viewed simultaneously to facilitate the accurate identification of neuroanatomical boundaries. Hippocampal tracing in each hemisphere began at the indentation of the hippocampal sulcus, or the most medial point of the hippocampus in the coronal plane. The alveus of the hippocampus was used as the superior boundary and the white matter of the parahippocampal gyrus as the inferior boundary. The inferior temporal horn of the lateral ventricle was used as the lateral boundary and the ambient cistern as the medial boundary. Hippocampal tracing was continued posteriorly until hippocampal gray matter formed an oval mass medial to the atrium of the lateral ventricles. Bilateral hippocampi were traced three times, twice by a single graduate student tracer and once by an experienced postdoctoral tracer. The average measure intraclass coefficient (ICC) was 0.88 with a 95% confidence interval of 0.77–0.94 [*F*_(22, 66)_ = 8.03, *p* < 0.001] between all three tracings. Subsequently, since only two tracers were used a thresholded mask was created using only the voxels that were chosen in all three tracings. Volume data was extracted, using FSL utilities, from all masks and entered into SPSS.

##### Anatomical definition of the amygdala

**Figure 1:**
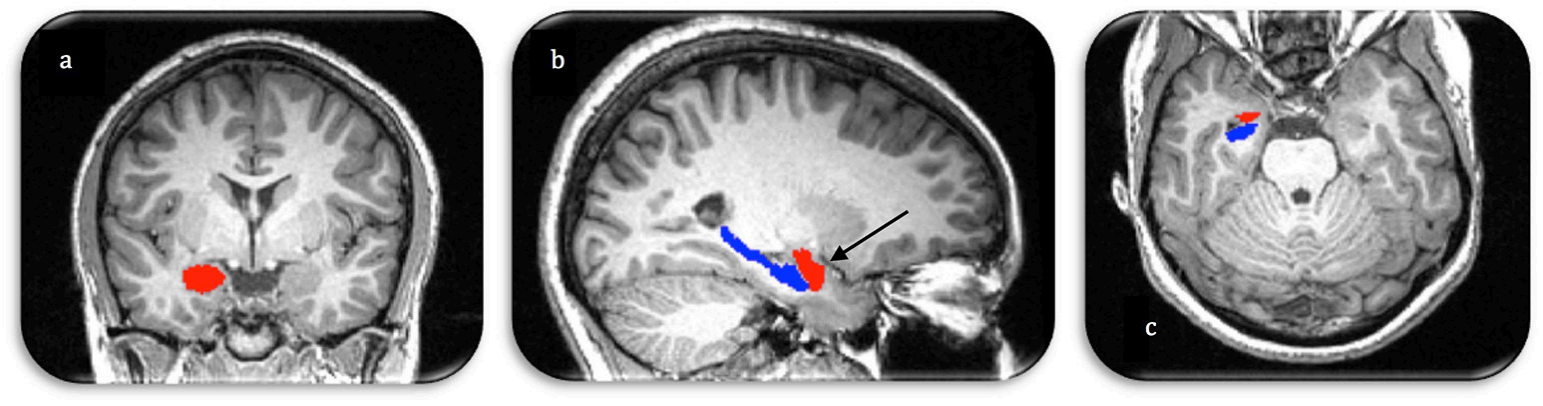
**Manual segmentation of the right amygdala (red) and hippocampus (blue) in three image planes (A. coronal, B. sagittal, C. axial)**. Arrow demonstrates the anterior boundary of the amygdala as visualized in all three orthogonal planes.

Separate left and right amygdala masks were hand-drawn onto each participant's T1-weighted image in the coronal plane. The amygdala was demarcated by superior, inferior, medial, and lateral boundaries and traced in the medial temporal lobe. The anterior boundary was defined as the slice that is considered to be amygdala as viewed in all three orthogonal slices (see Figure 1). The superior boundary of the amygdala was defined as the CSF within the temporal horn of the lateral ventricle for more anterior slices, while the visible gray-white matter boundary served as the superior border in more posterior slices. CSF defined the dorsomedial boundary, while the lateral boundary was defined as the border between amygdala gray matter and parahippocampal white matter. In anterior coronal slices, the inferior boundary was first demarcated by parahippocampal white matter and extended dorso-medially until the line connected with CSF. As the amygdala moved above the hippocampal gray matter in posterior slices, the inferior boundary was traced along the white matter strand of the alveus. Three different tracers (two graduate student tracers, one experienced postdoctoral tracer) traced bilateral amygdalae. An additional tracer was added to the amygdala tracing given that the amygdala is a smaller structure and more variability in measurement was expected. The average measure ICC was .80 with a 95% confidence interval of 0.62–0.89 [*F*_(22, 110)_ = 4.76 2, *p* < 0.001] between all three tracers. A thresholded mask was then created including all voxels that were chosen in *at least* 2 out of the 3 tracings to address variability in measurement. This majority voting procedure for manual tracing has been shown to be effective in a number of contexts (Aljabar et al., 2009). Volume data was extracted, using FSL utilities, from all masks and entered into SPSS.

### Statistical analyses

#### Analysis of automated segmentation performance

Comparisons of the automated and manual segmentations were done to explore differences in segmentation. The automated segmentation methods were compared to manual tracing using the following criteria from Morey et al. (2009): (1) percent volume overlap or Dice's coefficient, (2) percent volume difference and (3) correlation between automated measures and manual tracing. Given that the current study uses the volumetric output to measure the association between family aggression exposure and brain volume, percent volume difference is the most important outcome measure. Percent volume difference is defined as the absolute volume difference between two measures of the same structure divided by the mean volume of both segmentations and multiplied by 100 (Equation 1). This calculation, unlike overlap percentage, is insensitive to the spatial shift of the segmentations.

(1) $$\begin{array}{l} D\left(S_{1},S_{2}\right)=\frac{\left|S1-S2\right|}{\left(\frac{S1+S2}{2}\right)}\times 100 \end{array}$$

Percent volume overlap was calculated by means of FSL v5.0.8 (http://fsl.fmrib.ox.ac.uk/fsl/fslwiki/) library functions flsmaths and fslstats also used by Morey et al. (2009). Percent overlap is defined as the volume of the intersection of the two segmentations (*S*_1_ and *S*_2_), divided by the mean volume of these same segmentations, multiplied by 100 (Equation 2). Segmented labels from FIRST were extracted using fslmaths.

(2) $$\begin{array}{l} O\left(S_{1},S_{2}\right)=\frac{\left|S1\cap S2\right|}{\left(\frac{S1+S2}{2}\right)}\times 100 \end{array}$$

Percent overlap could not be calculated with the NeuroQuant segmentations because the output does not include a segmentation mask. Two-tailed bivariate pearson's correlations were calculated to test the relationship between the extracted volume data from each segmentation method in SPSS. A strong correlation (0.8 or above) indicates small volumes for small structures and large volumes for large structures.

#### Analysis of segmentations and behavioral measures

Multivariable linear regressions were run assessing the relationship between family aggression exposure and: (1) left hippocampus, (2) right hippocampus, (3) left amygdala, and (4) right amygdala, each with three different segmentation methods (manual, FSL, and NeuroQuant). Thus, 12 multivariable linear regressions were run, all accounting for age, gender, and total brain volume. Socioeconomic status, operationalized as family income, was tested as an additional covariate, but did not affect the results and was subsequently dropped.

## Results

### Automated segmentation performance

Table 1 summarizes bilateral hippocampi and amygdalae mean volumes obtained by manual, FSL and NeuroQuant segmentation methods. A one-way ANOVA found that for all four structures mean volumes significantly differed between the three analysis methods (*p* < 0.001). NeuroQuant segmentations were the largest for all structures, and were significantly larger than both FSL and manual segmentation. *Post-hoc* analyses revealed that for bilateral hippocampi, NeuroQuant segmentations were significantly larger than FSL segmentations, which in turn were significantly larger than manual tracing segmentations. For bilateral amygdalae, no difference between the manual tracing and FSL segmentations was found. However, the NeuroQuant segmentations were significantly bigger than both the FSL and manual segmentations.

**Table 1:** **Means, standard deviations, and comparison of means between manual and automated segmentations**.

	**Manual segmentation (mm^3^)**	**FSL segmentation (mm^3^)**	**NeuroQuant segmentation (mm^3^)**	**ANOVA**
Left Amygdala, mean (*SD*)	1222 (263)	1268 (207)	2447 (244)	*F*(2, 66) = 191.2, *p* < 0.001
Right Amygdala, mean (*SD*)	1193 (282)	1330 (241)	2326 (302)	*F*(2, 66) = 117.23, *p* < 0.001
Left Hippocampus, mean (*SD*)	1913 (329)	3854 (418)	4546 (432)	*F*(2, 66) = 248.7, *p* < 0.001
Right Hippocampus, mean (*SD*)	1844 (282)	3890 (425)	4745 (384)	*F*(2, 66) = 368.7, *p* < 0.001

Table 2 shows the results of the similarity/discrepancy analysis between the manual, FSL, and NeuroQuant methods for all structures. Percent overlap could only be calculated for FSL vs. manual segmentation, but as explained above, percent difference is more important for the purposes of the current analyses. Overlap percentages were higher for manual vs. FSL segmentations of bilateral amygdalae, than bilateral hippocampi. Overlap indicates spatial similarity between segmentations, thus the FSL and manual segmentations of bilateral amygdalae indicate a similar location in the brain, and less similar location for bilateral hippocampi. Similarly, lower percent volume difference was found in amygdalae segmentations than hippocampi segmentations for manual and FSL segmentations. Percent difference accounts for the number of voxels (mm^3^) that differ between segmentations. Therefore, a higher percent difference indicates less similarity between segmentations. The larger percent difference in hippocampal volumes may be due to FSL segmentations being significantly larger overall than manual segmentations.

**Table 2:** **Summary of automated segmentation performance, percent volume overlap, percent volume difference, and Pearson's correlations between automated and manual segmentations**.

	**Left amygdala *M (± SD)***	**Right amygdala *M (± SD)***	**Left hippocampus *M (± SD)***	**Right hippocampus *M (± SD)***
**MANUAL VS. FSL SEGMENTATIONS**				
% Overlap	73% (± 13)	73% (± 14)	64% (± 7)	61% (± 6)
% Difference	20% (± 11)	22% (± 18)	67% (± 16)	71% (± 12)
Pearson Correlation	0.31	0.20	0.37	0.56^**^
**NEUROQUANT VS. FSL SEGMENTATIONS**				
% Overlap	–	–	–	–
% Difference	63% (± 18)	55% (± 21)	16% (± 9)	20% (± 10)
Pearson Correlation	–0.06	0.01	0.56^**^	0.60^**^
**MANUAL VS. NEUROQUANT SEGMENTATIONS**				
% Overlap	–	–	–	–
% Difference	68% (± 20)	65% (± 24)	82% (± 13)	89% (± 10)
Pearson Correlation	0.07	0.03	0.47^*^	0.53^**^

* *p < 0.05*,

** *p < 0.001*.

Percent difference between FSL and NeuroQuant segmentations was larger for bilateral amygdalae than bilateral hippocampi. The difference for bilateral hippocampi between FSL and NeuroQuant segmentations was the smallest difference between all segmentations (16–20%), meaning the volume accounted for by these two segmentations was the most similar. Percent difference between manual and NeuroQuant segmentations was large for bilateral amygdalae and hippocampi. The largest difference between any segmentations were between manual vs. NeuroQuant segmentations for bilateral hippocampi.

The only significant Pearson's correlations between segmentations occurred in the hippocampal segmentations, which suggests that the rank order of participants may be preserved across methods although absolute volume differs. Only right hippocampal segmentations were significantly correlated and were in the moderate range when comparing manual and FSL segmentations. Bilateral hippocampi segmentations were also moderately correlated between FSL and NeuroQuant segmentations and between manual and NeuroQuant segmentations.

### Associations between brain volume and family aggression exposure

#### FSL segmentations

**Figure 2:**
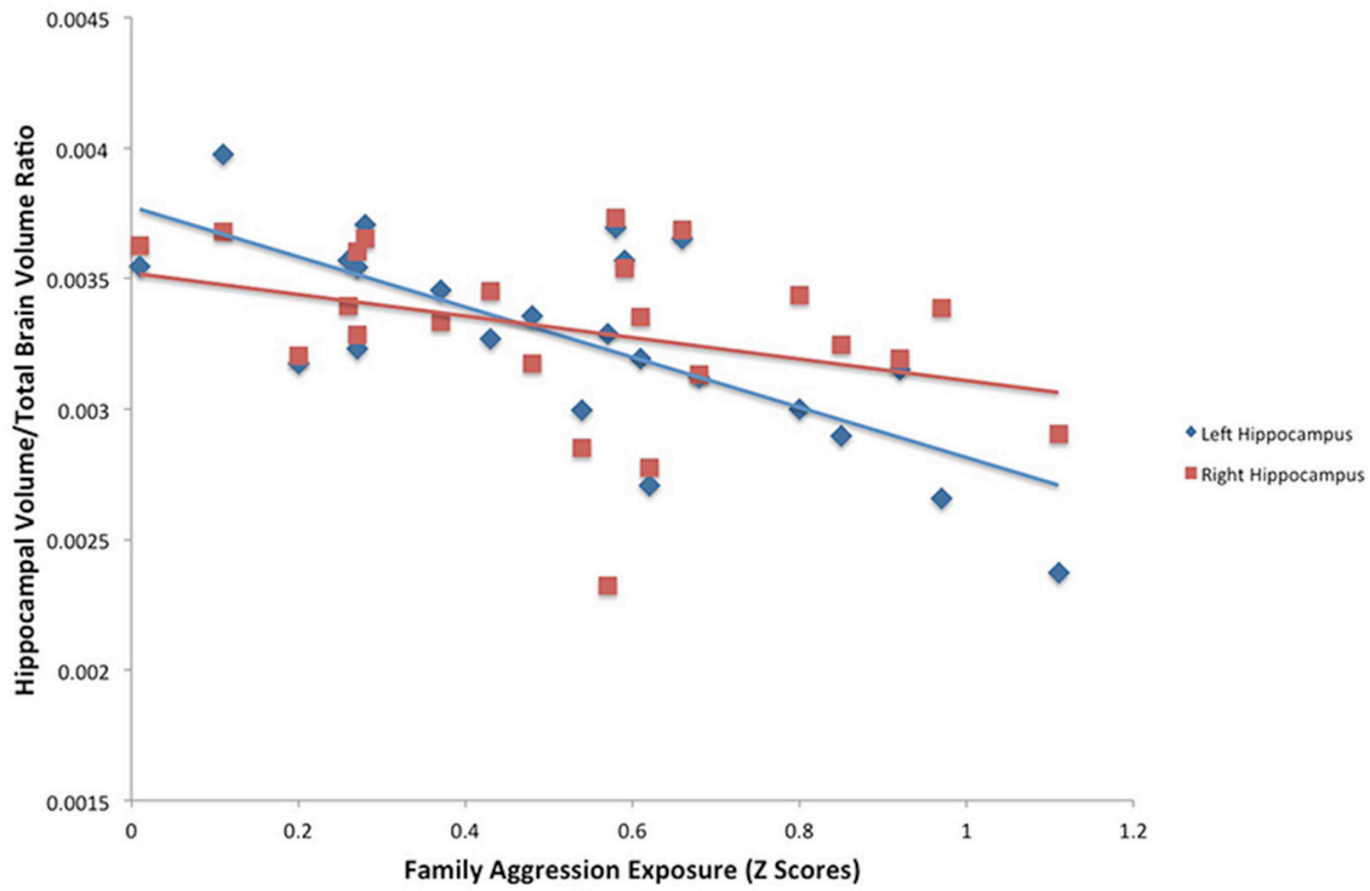
**Results of a partial correlation representing the relationship between family aggression exposure and bilateral hippocampal volume measured by FSL segmentation**. A significant negative correlation was found between bilateral hippocampal volume (left: β = −0.75, *p* < 0.001; right: β = −0.48, *p* < 0.05) and family aggression exposure accounting for total brain volume.

A significant relationship between exposure to family aggression and brain volume was found in bilateral hippocampi using the FSL segmentation estimates. As seen in Table 3, Figure 2, a significant negative relationship between left hippocampal volume and family aggression exposure was found. Similarly, a significant negative relationship between right hippocampal volume and family aggression exposure also was found. No other significant relationships were found between family aggression exposure and brain volume using FSL automated segmentations.

**Table 3:** **Separate multivariate linear regression analyses of family aggression exposure and manual and automated bilateral hippocampi and amygdalae segmentations, adjusting for age, gender and total brain volume**.

**Segmentation**	**Structure**	**β**
FSL	L.HC	−0.75^**^
	R.HC	−0.48^*^
	L.Amyg	−0.18
	R.Amyg	−0.20
Manual		
	L.HC	0.04
	R.HC	−0.06
	L.Amyg	0.87
	R.Amyg	0.47^*^
NeuroQuant	L.HC	−0.09
	R.HC	−0.13
	L.Amyg	−0.03
	R.Amyg	−0.15

* *p < 0.05*,

** *p < 0.001 L.HC, Left Hippocampus; R.HC, Right Hippocampus; L.Amyg, Left Amygdala; R.Amyg, Right Amygdala*.

#### Manual segmentations

**Figure 3:**
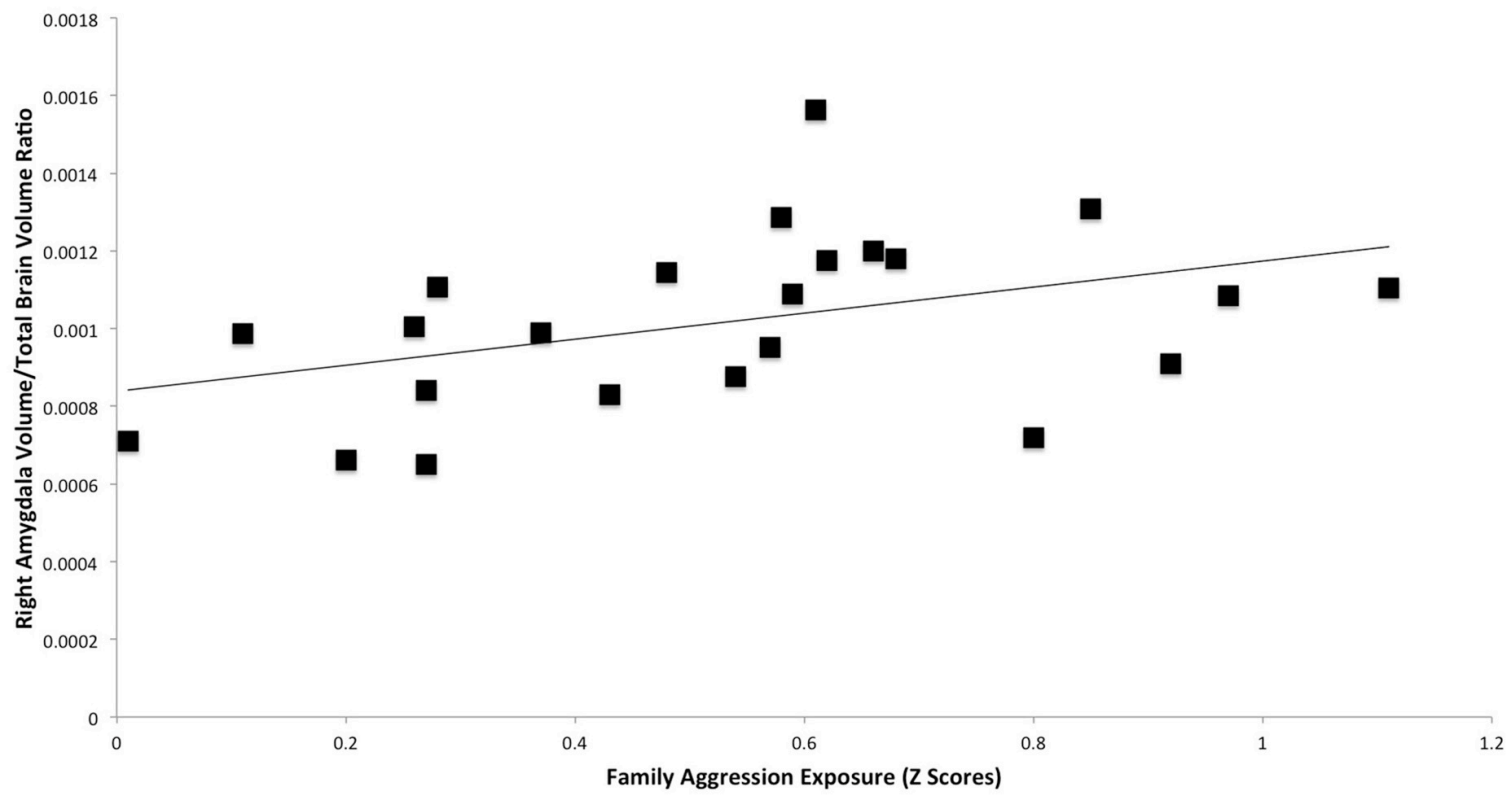
**Results of a partial correlation representing the relationship between family aggression exposure and right amygdala volume measured by manual segmentation**. A significant positive correlation was found between right amygdala volume (β = 0.47, *p* < 0.05) and family aggression exposure accounting for total brain volume.

A significant positive relationship was found between right amygdala volume and family aggression exposure using manual segmentations (Table 3, Figure 3). No other relationships between family aggression exposure and amygdala or hippocampal volume were found using the manual segmentations.

#### NeuroQuant segmentations

No significant relationships between exposure to family aggression and brain volume were found in bilateral amygdalae or hippocampi using the NeuroQuant segmentation estimates.

## Discussion

The current study sought to investigate the relationship between family aggression exposure and brain volume using three different segmentation estimates of bilateral amygdalae and hippocampi. Results indicate inconsistencies among all three methods, and notably different associations between family aggression exposure and brain volume were found depending on the segmentation method used. The hypothesized relationship between family aggression exposure and amygdalae volume was found but only with manual segmentation estimates of the right amygdala. As hypothesized, smaller bilateral hippocampal volume was found to be associated with more family aggression exposure, but only when using the FSL segmentations of the hippocampus. Therefore, although both results are in line with previous literature, they do not emerge across all three methods.

Inconsistencies were also found between methods. Manual tracing segmentations were found to be the smallest of the segmentations for bilateral hippocampi and amygdalae. Manual segmentation is considered to be the “gold standard” for structural analysis, as it is based on the individual subject's brain anatomy (Morey et al., 2009). Therefore, smaller manual segmentations may be indicative of the developmental trajectory of the amygdalae during adolescence.

The automated segmentations (FSL and NeuroQuant) were found to be the most similar in terms of volume for bilateral hippocampi. FSL and manual segmentations, however, were found to be the most similar for bilateral amygdalae. The largest difference was found between NeuroQuant and manual bilateral hippocampal segmentations. The percent difference between NeuroQuant segmentations and manual segmentations for bilateral hippocampi was close to 90%. Percent difference for all amygdalae segmentations was between 20 and 68% indicating poor volume similarity between all amygdalae segmentations. Previous studies comparing methodologies similarly found better overlap between methods for hippocampal estimates than amygdala estimates (Morey et al., 2009). However, the discrepancies between manual segmentations and NeuroQuant segmentations are larger than previous investigations (Heckemann et al., 2006; Jatzko et al., 2006; Barnes et al., 2008; Powell et al., 2008; Aljabar et al., 2009; Artaechevarria et al., 2009; Morey et al., 2009; Lehmann et al., 2010; Fellhauer et al., 2015), regardless of using an age-matched atlas for adolescents in NeuroQuant segmentations. The differences between methods may be due to many factors. First of all, manually traced and FSL segmentations used AC–PC aligned images and similar preprocessing procedures, whereas NeuroQuant did not use AC–PC aligned images and has different preprocessing steps. However, although in some instances FSL and manual segmentations were the most similar (bilateral amygdalae), FSL and NeuroQuant segmentations were most similar for bilateral hippocampi. An additional source of variation in segmentation is due to differential registration algorithms to the respective atlases used in each of the automated procedures. FSL FIRST registers each individual's brain to MNI152 space at 1 mm resolution, whereas NeuroQuant uses a dynamic atlas that has been validated with thousands of clinical age-matched scans. Although these differences exist between methods, each method has been used and published on its own, and differences between methods in these published papers have not been extensively discussed. The current study aimed to investigate if differences between segmentation methods that are all commonly used in the literature would lead to different results when used to investigate the same research question. Therefore, the methods were optimized for their own procedures and not for ease of comparison, given our interest in examining inherent differences in these methods.

### Brain volume and family aggression exposure

#### Manual segmentations

Larger right amygdala volume was found to be associated with higher levels of family aggression exposure using manually traced estimates of amygdala volume. No other significant relationships between bilateral hippocampi or left amygdala and family aggression exposure were found. If manual tracing is taken as the “gold standard” for brain volume segmentation, these findings are consistent with previous research indicating that early childhood adversity may be linked with chronic overactivation of the amygdala and atypical expansion during adolescence. However, the current investigation also showed that different methodologies led to different substantive results in the adolescent brain.

#### FSL segmentations

Smaller left and right hippocampal volumes were found to be associated with greater family aggression exposure using FSL segmentation estimates of hippocampal volume. This finding is consistent with previous research indicating an association between early childhood adversity and smaller hippocampal volume measured in adulthood. Previous studies have questioned whether this association occurs after atrophy throughout childhood and adolescence, or if this association can be measured before adulthood. The current study indicates that this association may be measurable in late adolescence, at least if the FSL segmentations are used.

#### NeuroQuant segmentations

No relationship between early life family aggression exposure and brain volume was found using the NeuroQuant segmentation estimates. Similar to the FSL segmentations, the NeuroQuant segmentations were significantly larger than the manual tracing segmentations.

## Conclusion

The most striking result of the current investigation is that the choice of different segmentation methods led to different associations with a variable of interest, namely family aggression exposure. Although limitations in the current study exist, the fact that each result may have been found on its own if we had not compared across methodologies points to a potentially significant problem in the structural MRI literature. The current investigation found associations between family aggression exposure and both amygdala and hippocampal volumes in the adolescent brain. However, these results should be interpreted cautiously given the discrepancies between methods that emerged. Although three significant findings out of 12 is above chance-level, it nevertheless raises the issue of multiple comparisons. Our results suggest that, with an adolescent population in particular, automated methods for subcortical brain volume estimation may be unreliable.

This study had several limitations. First, although the family aggression measures assessed conflict behavior over the previous year, it is possible that aggression occurred earlier and with greater chronicity in some study participants. Second, no baseline MRI scans exist to account for individual differences in brain volume before the event of aggression exposure. Third, although a sample size of 23 is not unusual in the neuroimaging literature, it may offer limited statistical power. Additionally, although we controlled for chronological age in all analyses, we did not have a measurement of pubertal stage, which has been shown to also be correlated with neural development (Blakemore et al., 2010). Future investigations would benefit from longitudinal scanning data and more precise measures of family aggression in early childhood. Similarly, investigating alternate automated segmentation software may be beneficial given the amount of time and effort needed for manual segmentation methods.

Despite its limitations, this paper makes a contribution to the literature as an example of the disparate results given in structural MRI analysis depending on the choice of segmentation method used. Similar papers have been published for cortical thickness (Martínez et al., 2015) and voxel-based morphometry (Rajagopalan and Pioro, 2015), suggesting that the choice of segmentation method may determine disparate associations with a variable of interest. Strengths of the current investigation include our family aggression measure which was assessed in multiple domains (marital and parent-child) with data from multiple reporters (both parents and the youth). Many studies of childhood adversity have relied on retrospective self-report, so our collection of multi-rater family aggression data during childhood is a strength of the study. Also, other studies comparing manual vs. automated segmentation approaches have often compared data from only a subset of participants, or have used manually traced images from only one tracer. We manually traced all participants' subcortical structures, and used multiple tracers, with good interrater reliability between tracers.

In conclusion, these results suggest that the choice of methods in any given structural analytic investigation can drastically influence results. The current study is a caution to both researchers and readers of structural neuroimaging investigations to be skeptical of the measures used for a specific population and a specific research question. It also suggests that the field of structural neuroimaging needs to become more rigorous and systematic in the ways in which methods are chosen and carried out. Additionally, automated segmentation approaches, which are widely used, need to be further refined and perfected in order to capture more discrete individual differences in neurobiology.

## Author contributions

All co-authors contributed substantively to this work and agree on its content. HL designed the hypotheses and research questions, analyzed data, and wrote the paper. DS supervised the project, helped with design and hypotheses, oversaw data analysis, collected MRI data, and reviewed the manuscript. GM, JK supervised the project and revised the manuscript. SG, MS and AT analyzed data, traced structures, and helped to write the manuscript. LD gathered MRI data, helped with data analysis, and revised the manuscript.

## Funding

This study was funded by NIH-NICHD NRSA Post-doctoral Fellowship F32 HD63255 and NIH-NICHD R01 HD046807.

### Conflict of interest statement

The authors declare that the research was conducted in the absence of any commercial or financial relationships that could be construed as a potential conflict of interest. The reviewer JSG and handling Editor declared their shared affiliation, and the handling Editor states that the process nevertheless met the standards of a fair and objective review.

## References

[B1] Adolphs R. Tranel D. Damasio H. Damasio A. R. (1994). Impaired recognition of emotion in facial expressions following bilateral damage to the human amygdala. Nature 372, 669–672. 10.1038/372669a0 7990957

[B2] Aljabar P. Heckemann R. A. Hammers A. Hajnal J. V. Rueckert D. (2009). Multi-atlas based segmentation of brain images: atlas selection and its effect on accuracy. Neuroimage 46, 726–738. 10.1016/j.neuroimage.2009.02.018 19245840

[B3] Allen J. S. Emmorey K. Bruss J. Damasio H. (2008). Morphology of the insula in relation to hearing status and sign language experience. J. Neurosci. 28, 11900–11905. 10.1523/JNEUROSCI.3141-08.2008 19005055 PMC2606108

[B4] Artaechevarria X. Muñoz-Barrutia A. Ortiz-de Solorzano C. (2009). Combination strategies in multi-atlas image segmentation: application to brain MR data. IEEE Trans. Med. Imaging 28, 1266–1277. 10.1109/TMI.2009.2014372 19228554

[B5] Barnes J. Foster J. Boyes R. G. Pepple T. Moore E. K. Schott J. M.. (2008). A comparison of methods for the automated calculation of volumes and atrophy rates in the hippocampus. Neuroimage 40, 1655–1671. 10.1016/j.neuroimage.2008.01.012 18353687

[B6] Blakemore S.-J. Burnett S. Dahl R. E. (2010). The role of puberty in the developing adolescent brain. Hum. Brain Mapp. 31, 926–933. 10.1002/hbm.21052 20496383 PMC3410522

[B7] Bremner J. D. Randall P. Vermetten E. Staib L. Bronen R. A. Mazure C.. (1997). Magnetic resonance imaging-based measurement of hippocampal volume in posttraumatic stress disorder related to childhood physical and sexual abuse–a preliminary report. Biol. Psychiatry 41, 23–32. 8988792 10.1016/s0006-3223(96)00162-x PMC3229101

[B8] Cabezas M. Oliver A. Lladó X. Freixenet J. Cuadra M. B. (2011). A review of atlas-based segmentation for magnetic resonance brain images. Comput. Methods Programs Biomed. 104, 158–177. 10.1016/j.cmpb.2011.07.015 21871688

[B9] Carrion V. G. Weems C. F. Eliez S. Patwardhan A. Brown W. Ray R. D.. (2001). Attenuation of frontal asymmetry in pediatric posttraumatic stress disorder. Biol. Psychiatry 50, 943–951. 10.1016/S0006-3223(01)01218-5 11750890

[B10] Casey B. J. Jones R. M. Hare T. A. (2008). The adolescent brain. Ann. N. Y. Acad. Sci. 1124, 111–126. 10.1196/annals.1440.010 18400927 PMC2475802

[B11] Davis M. Whalen P. J. (2001). The amygdala: vigilance and emotion. Mol. Psychiatry 6, 13–34. 10.1038/sj.mp.4000812 11244481

[B12] De Bellis M. D. (2001). Developmental traumatology: the psychobiological development of maltreated children and its implications for research, treatment, and policy. Dev. Psychopathol. 13, 539–564. 10.1017/S0954579401003078 11523847

[B13] De Bellis M. D. Keshavan M. S. Shifflett H. Iyengar S. Beers S. R. Hall J.. (2002). Brain structures in pediatric maltreatment-related posttraumatic stress disorder: a sociodemographically matched study. Biol. Psychiatry 52, 1066–1078. 10.1016/S0006-3223(02)01459-2 12460690

[B14] Dewey J. Hana G. Russell T. Price J. McCaffrey D. Harezlak J.. (2010). Reliability and validity of MRI-based automated volumetry software relative to auto-assisted manual measurement of subcortical structures in HIV-infected patients from a multisite study. Neuroimage 51, 1334–1344. 10.1016/j.neuroimage.2010.03.033 20338250 PMC2884380

[B15] Doring T. M. Kubo T. T. Cruz L. C. H. Juruena M. F. Fainberg J. Domingues R. C.. (2011). Evaluation of hippocampal volume based on MR imaging in patients with bipolar affective disorder applying manual and automatic segmentation techniques. J. Magn. Reson. Imaging 33, 565–572. 10.1002/jmri.22473 21563239

[B16] Driessen M. Herrmann J. Stahl K. Zwaan M. Meier S. Hill A.. (2000). Magnetic resonance imaging volumes of the hippocampus and the amygdala in women with borderline personality disorder and early traumatization. Arch. Gen. Psychiatry 57, 1115–1122. 10.1001/archpsyc.57.12.1115 11115325

[B17] Felitti V. J. Anda R. F. Nordenberg D. Williamson D. F. Spitz A. M. Edwards V.. (1998). Relationship of childhood abuse and household dysfunction to many of the leading causes of death in adults: the Adverse Childhood Experiences (ACE) Study. Am. J. Prev. Med. 14, 245–258. 10.1016/S0749-3797(98)00017-8 9635069

[B18] Fellhauer I. Zöllner F. G. Schröder J. Degen C. Kong L. Essig M.. (2015). Comparison of automated brain segmentation using a brain phantom and patients with early Alzheimer's dementia or mild cognitive impairment. Psychiatry Res. 233, 299–305. 10.1016/j.pscychresns.2016.08.003 26211622

[B19] Fischl B. Salat D. H. Busa E. Albert M. Dieterich M. Haselgrove C.. (2002). Whole brain segmentation: automated labeling of neuroanatomical structures in the human brain. Neuron 33, 341–355. 10.1016/S0896-6273(02)00569-X 11832223

[B20] Frodl T. Reinhold E. Koutsouleris N. Reiser M. Meisenzahl E. M. (2010). Interaction of childhood stress with hippocampus and prefrontal cortex volume reduction in major depression. J. Psychiatr. Res. 44, 799–807. 10.1016/j.jpsychires.2010.01.006 20122698

[B21] Hamann S. B. Ely T. D. Hoffman J. M. Kilts C. D. (2002). Ecstasy and agony: activation of the human amygdala in positive and negative emotion. Psychol. Sci. 13, 135–141. 10.1111/1467-9280.00425 11933997

[B22] Han X. Fischl B. (2007). Atlas renormalization for improved brain MR image segmentation across scanner platforms. IEEE Trans. Med. Imaging 26, 479–486. 10.1109/TMI.2007.893282 17427735

[B23] Heckemann R. A. Hajnal J. V. Aljabar P. Rueckert D. Hammers A. (2006). Automatic anatomical brain MRI segmentation combining label propogation and decision fusion. Neuroimage 1, 115–126. 10.1109/TMI.2007.893282 16860573

[B24] Heim C. Newport D. J. Wagner D. Wilcox M. M. Miller A. H. Nemeroff C. B. (2002). The role of early adverse experience and adulthood stress in the prediction of neuroendocrine stress reactivity in women: a multiple regression analysis. Depress. Anxiety 15, 117–125. 10.1002/da.10015 12001180

[B25] Jatzko A. Röthenhofer S. Schmitt A. Gaser C. Demirakca T. Weber-Fahr W.. (2006). Hippocampal volume in chronic posttraumatic stress disorder (PTSD): MRI study using two different evaluation methods. J. Affect. Disord. 94, 121–126. 10.1016/j.jad.2006.03.010 16701903

[B26] Keenan P. A. Jacobson M. W. Soleymani R. M. Mayes M. D. Stress M. E. Yaldoo D. T. (1996). The effect on memory of chronic prednisone treatment in patients with systemic disease. Neurology 47, 1396–1402. 10.1212/WNL.47.6.1396 8960717

[B27] Kovacevic S. Rafii M. S. Brewer J. B. (2009). High-throughput, fully-automated volumetry for prediction of MMSE and CDR decline in mild cognitive impairment. Alzheimer Dis. Assoc. Disord. 23, 139. 10.1097/WAD.0b013e318192e745 19474571 PMC2688740

[B28] Lehmann M. Douiri A. Kim L. G. Modat M. Chan D. Ourselin S.. (2010). Atrophy patterns in Alzheimer's disease and semantic dementia: a comparison of FreeSurfer and manual volumetric measurements. Neuroimage 49, 2264–2274. 10.1016/j.neuroimage.2009.10.056 19874902

[B29] Lobaugh N. J. Gibson E. Taylor M. J. (2006). Children recruit distinct neural systems for implicit emotional face processing. Neuroreport 17, 215–219. 10.1097/01.wnr.0000198946.00445.2f 16407774

[B30] Lupien S. J. Fiocco A. Wan N. Maheu F. Lord C. Schramek T.. (2005). Stress hormones and human memory function across the lifespan. Psychoneuroendocrinology 30, 225–242. 10.1016/j.psyneuen.2004.08.003 15511597

[B31] Margolin G. Gordis E. B. (2000). The effects of family and community violence on children. Annu. Rev. Psychol. 51, 445–479. 10.1146/annurev.psych.51.1.445 10751978

[B32] Margolin G. Gordis E. B. John R. S. (2001). Coparenting: a link between marital conflict and parenting in two parent families. J. Fam. Psychol. 15, 3–21. 10.1037/0893-3200.15.1.3 11322083

[B33] Margolin G. John R. S. Foo L. (1998). Interactive and unique risk factors for husbands' emotional and physical abuse of their wives. J. Fam. Violence 13, 315–344. 10.1023/A:102288051836

[B34] Margolin G. Vickerman K. A. Oliver P. H. Gordis E. B. (2010).Violence exposure in multiple interpersonal domains: cumulative and differential effects. J. Adolesc. Health 47, 198–205. 10.1016/j.jadohealth.2010.01.020 20638013 PMC2907247

[B35] Markowitsch H. J. Pritzel M. (1985). The neuropathology of amnesia. Prog. Neurobiol. 25, 189–287. 10.1016/0301-0082(85)90016-4 4089179

[B36] Martínez K. Madsen S. K. Joshi A. A. Joshi S. H. Román F. J. Villalon-Reina J.. (2015). Reproducibility of brain-cognition relationships using three cortical surface-based protocols: an exhaustive analysis based on cortical thickness. Hum. Brain Mapp. 6, 3227–3245. 10.1002/hbm.22843 26032714 PMC6868955

[B37] Mehta M. A. Golembo N. I. Nosarti C. Colvert E. Mota A. Williams S. C.. (2009). Amygdala, hippocampal and corpus callosum size following severe early institutional deprivation: the English and Romanian Adoptees study pilot. J. Child Psychol. Psychiatry 50, 943–951. 10.1111/j.1469-7610.2009.02084.x 19457047

[B38] Morey R. A. Petty C. M. Xu Y. Hayes J. P. Wagner H. R. Lewis D. V.. (2009). A comparison of automated segmentation and manual tracing for quantifying hippocampal and amygdala volumes. Neuroimage 45, 855–866. 10.1016/j.neuroimage.2008.12.033 19162198 PMC2714773

[B39] Mulder E. R. de Jong R. A. Knol D. L. van Schijndel R. A. Cover K. S. Visser P. J.. (2014). Hippocampal volume change measurement: quantitative assessment of the reproducibility of expert manual outlining and the automated methods FreeSurfer and FIRST. Neuroimage 92, 169–181. 10.1016/j.neuroimage.2014.01.058 24521851

[B40] Narr K. L. Thompson P. M. Szesko P. Robinson D. Jang S. Woods R. P.. (2004). Regional specificity of hippocampal volume reductions in first episode schizophrenia. Neuroimage 21, 1563–1575. 10.1016/j.neuroimage.2003.11.011 15050580

[B41] Newcomer J. W. Craft S. Hershey T. Askins K. Bardgett M. E. (1994). Glucocorticoid-induced impairment in declarative memory performance in adult humans. J. Neurosci. 14, 2047–2053. 8198631 10.1523/JNEUROSCI.14-04-02047.1994 PMC6577116

[B42] Patenaude B. Smith S. M. Kennedy D. Jenkinson M. (2011). A Bayesian model of shape and appearance for subcortical brain Neuroimage 56, 907–922. 10.1016/j.neuroimage.2011.02.046 21352927 PMC3417233

[B43] Perry B. D. (2001). The neurodevelopmental impact of violence in childhood. Chapter 18, in Textbook of Child and Adolescent Forensic Psychiatry, Eds Schetky D. Benedek E. P. (Washington, DC: American Psychiatric Press, Inc.,), 221–238.

[B44] Powell S. Magnotta V. A. Johnson H. Jammalamadaka V. K. Pierson R. Andreasen N. C. (2008). Registration and machine learning-based automated segmentation of subcortical and cerebellar brain structures. Neuroimage 39, 238–247. 10.1016/j.neuroimage.2007.05.063 17904870 PMC2253948

[B45] Rajagopalan V. Pioro E. P. (2015). Disparate voxel based morphometry (VBM) results between SPM and FSL softwares in ALS patients with frontotemporal dementia: which VBM results to consider. Biomed. Central Neurol. 15–32. 10.1186/s12883-015-0274-8 25879588 PMC4371611

[B46] Rao H. Betancourt L. Giannetta J. M. Brodsky N. L. Korczykowski M. Avants B.. (2010). Early parental care is important for hippocampal maturation: evidence from brain morphology in humans. Neuroimage 49, 1144–1150. 10.1016/j.neuroimage.2009.07.003 19595774 PMC2764790

[B47] Repetti R. L. Taylor S. E. Seeman T. E. (2002). Risky families: family social environments and the mental and physical health of offspring. Psychol. Bull. 128:330. 10.1037/0033-2909.128.2.330 11931522

[B48] Sanchez-Benavides G. Gomez-Anson B. Sainz A Vives, Y. Deflino M. Pena-Casanova J. (2010). Manual validation of FreeSurfer's automated hippocampal segmentation in normal aging, mild cognitive impairment, and Alzheimer Disease subjects. Psychiatry Res. 181, 219–225. 10.1016/j.pscychresns.2009.10.011 20153146

[B49] Schmahl C. G. Vermetten E. Elzinga B. M. Bremner D. J. (2003). Magnetic resonance imaging of hippocampal and amygdala volume in women with childhood abuse and borderline personality disorder. Psychiatry Res. 122, 193–198. 10.1016/S0925-4927(03)00023-4 12694893

[B50] Seixas F. L. Saade D. C. M. Conci A. de Souza A. S. Tovar-Moll F. Bramatti I. (2010). Anatomical brain MRI segmentation methods: volumetric assessment of the hippocampus, in IWSSIP 2010–17th International Conference on Systems, Signals and Image Processing. (Rio de Janiero: EdUFF), 247–250.

[B51] Shen L. Saykin A. J. Kim S. Firpi H. A. West J. D. Risacher S. L.. (2010). Comparison of manual and automated determination of hippocampal volumes in MCI and early AD. Brain Imaging Behav. 4, 86–95. 10.1007/s11682-010-9088-x 20454594 PMC2863347

[B52] Straus M. A. Hamby S. L. Finkelhor D. Moore D. W. Runyan D. (1998). Identification of child maltreatment with the parent child conflict tactics scales: development and psychometric data for a national sample of American parents. Child Abuse Neglect 22, 249–270. 10.1016/S0145-2134(97)00174-9 9589178

[B53] Teicher M. H. Samson J. A. Polcari A. McGreenery C. E. (2006). Sticks, stones, and hurtful words: relative effects of various forms of childhood maltreatment. Am. J. Psychiatry 163, 993–1000. 10.1176/appi.ajp.163.6.993 16741199

[B54] Thomas K. M. Drevets W. C. Dahl R. E. Ryan N. D. Birmaher B. Eccard C. H.. (2001). Amygdala response to fearful faces in anxious and depressed children. Arch. Gen. Psychiatry 58, 1057–1063. 10.1001/archpsyc.58.11.1057 11695953

[B55] Tottenham N. Hare T. A. Casey B. J. (2009a). A developmental perspective on human amygdala function, in The Human Amygdala, eds Phelps E. Whalen P. (New York, NY: Guilford Press), 107–117.

[B56] Tottenham N. Hare T. A. Quinn B. T. McCarry T. W. Nurse M. Gilhooly T.. (2009b). Prolonged institutional rearing is associated with atypically large amygdala volume and emotion regulation difficulties. Dev. Sci. 13, 46–61. 10.1111/j.1467-7687.2009.00852.x 20121862 PMC2817950

[B57] Tottenham N. Sheridan M. A. (2010). A review of adversity, the amygdala and the hippocampus: a consideration of developmental timing. Front. Hum. Neurosci. 8:68. 10.3389/neuro.09.068.2009 20161700 PMC2813726

[B58] U. S. Census Bureau (2010). Cost of Living Index_Selected Urban Areas, Annual Average: 2010. Available online at: http://www.census.gov/2010census/

[B59] U. S. Census Bureau (2011). Selected Economiccharacteristics 2007–2011 American Community Survey 5-year Estimates. Available online at: http://factfinder2.census.gov/faces/tableservices/jsf/pp./productview.xhtml?src_bkmk

[B60] Vyas A. Pillai A. G. Chattarji S. (2004). Recovery after chronic stress fails to reverse amygdaloid neuronal hypertrophy and enhanced anxiety-like behavior. Neuroscience 128, 667–673. 10.1016/j.neuroscience.2004.07.013 15464275

[B61] Wolkowitz O. M. Reus V. I. Weingartner H. Thompson K. Breier A. Doran A.. (1990). Cognitive effects of corticosteroids. Am. J. Psychiatry 147, 1297–1303. 2399996 10.1176/ajp.147.10.1297

[B62] Woon F. L. Hedges D. W. (2008). Hippocampal and amygdala volumes in children and adults with childhood maltreatment-related posttraumatic stress disorder: a meta-analysis. Hippocampus 18, 729–736. 10.1002/hipo.20437 18446827

[B63] Yang J. Hou C. Ma N. Liu J. Zhang Y. Zhou J.. (2007). Enriched environment treatment restores impaired hippocampal synaptic plasticity and cognitive deficits induced by prenatal chronic stress. Neurobiol. Learn. Mem. 87, 257–263. 10.1016/j.nlm.2006.09.001 17049888

