# Corrigendum: Associations between Family Adversity and Brain Volume in Adolescence: Manual vs. Automated Brain Segmentation Yields Different Results

**DOI:** 10.3389/fnins.2016.00555

**Published:** 2016-11-29

**Authors:** Hannah Lyden, Sarah I. Gimbel, Larissa Del Piero, A. Bryna Tsai, Matthew Sachs, Jonas T. Kaplan, Gayla Margolin, Darby Saxbe

**Affiliations:** ^1^Department of Psychology, University of Southern CaliforniaLos Angeles, CA, USA; ^2^Department of Psychology, Brain and Creativity Institute, University of Southern CaliforniaLos Angeles, CA, USA

**Keywords:** amygdala, hippocampus, methodology, family aggression, early life stress, adolescence

Reason for Corrigendum:

In the original article there was an error in the beta value reporting the association between left amygdala volume calculated with manual segmentation and family aggression exposure in early life. The correct version of Table 3 appears below.

**Table 3:** **Separate multivariate linear regression analyses of family aggression exposure manual and automated bilateral hippocampal and amygdala segmentations adjusting for age, gender, and total brain volume**.

**Segmentation**	**Structure**	**β**
**FSL**		
	L.HC	−0.75^**^
	R.HC	−0.48^*^
	L.Amyg	−0.18
	R.Amyg	−0.20
**MANUAL**		
	L.HC	0.04
	R.HC	−0.06
	L.Amyg	0.39^+^
	R.Amyg	0.47^*^
**NEUROQUANT**		
	L.HC	−0.09
	R.HC	−0.13
	L.Amyg	−0.03
	R.Amyg	−0.15

* *p < 0.05*,

** *p < 0.001*,

+ *p = 0.09. L.HC, Left Hippocampus; R.HC, Right Hippocampus, L.Amyg, Left Amygdala; R.Amyg, Right Amygdala. TBV Ratio, Ratio of each segmentation to total gray matter plus white matter. Bold values indicate significant values*.

In the “Results” section, sub section “Manual Segmentation,” the second sentence has been added stating the following: A positive relationship between left amygdala volume and family aggression exposure using manual segmentation was approaching significance (*b* = 0.39, *p* = 0.09). The authors sincerely apologize for the error. This correlation was not previously mentioned or discussed in the manuscript. Therefore, this error does not change the scientific conclusions of the paper in any way.

## Conflict of interest statement

The authors declare that the research was conducted in the absence of any commercial or financial relationships that could be construed as a potential conflict of interest.

