# Corrigendum: Associations between Family Adversity and Brain Volume in Adolescence: Manual vs. Automated Brain Segmentation Yields Different Results

**DOI:** 10.3389/fnins.2017.00458

**Published:** 2017-08-29

**Authors:** Hannah Lyden, Sarah I. Gimbel, Larissa Del Piero, A. Bryna Tsai, Matthew E. Sachs, Jonas T. Kaplan, Gayla Margolin, Darby Saxbe

**Affiliations:** ^1^Department of Psychology, University of Southern California Los Angeles, CA, United States; ^2^Department of Psychology, Brain and Creativity Institute, University of Southern California Los Angeles, CA, United States

**Keywords:** amygdala, hippocampus, methodology, family aggression, early life stress, adolescence

In the original article citations were accidentally omitted and sentences were written in error in the paragraphs titled: *Anatomical definition of the hippocampus* and *Anatomical definition of the amygdala*.

The paragraph titled *Anatomical definition of the amygdala* should read as follows (corrections italicized):

Separate left and right amygdala masks were hand-drawn onto each participant's T1-weighted image in the coronal *plane according to tracing procedures described by Allen et al. (2005) and Clewett et al. (2014). As described by Clewett et al. (2014) the amygdala was traced in the medial temporal lobe. The anterior boundary was defined as the slice that is considered to be amygdala as viewed in all three orthogonal slices (see **Figure 1**). We defined the superior boundary of the amygdala as the CSF within the temporal horn of the lateral ventricle for more anterior slices. In more posterior slices, we used the visible gray-white matter boundary as the superior border. CSF defined the dorsomedial boundary. We defined the border between amygdala gray matter and parahippocampal white matter as the lateral boundary. In anterior coronal slices, we defined the inferior boundary by parahippocampal white matter and then extended the line dorsomedially until it connected with CSF. The inferior boundary was traced along the white matter strand of the alveus*. Three different tracers (two graduate student tracers, one experienced postdoctoral tracer) traced bilateral amygdalae. An additional tracer was added to the amygdala tracing given that the amygdala is a smaller structure and more variability in measurement was expected. The average measure ICC was 0.80 with a 95% confidence interval of 0.62–0.89 [*F*_(22, 110)_ = 4.76 2, *p* < 0.001] between all three tracers. A thresholded mask was then created including all voxels that were chosen in at least 2 out of the 3 tracings to address variability in measurement. This majority voting procedure for manual tracing has been shown to be effective in a number of contexts (Aljabar et al., 2009). Volume data was extracted, using FSL utilities, from all masks and entered into SPSS.

The paragraph titled *Anatomical definition of the hippocampus* should read as follows (corrections italicized):

The neuroanatomical criteria chosen for hippocampal delineation were taken from existing protocols (Narr et al., 2004; Nicolson et al., 2006). The hippocampi were traced in coronal brain slices from anterior to posterior, using fslview tools. All three (sagittal, coronal, and axial) planes were viewed simultaneously to facilitate the accurate identification of neuroanatomical boundaries. *As described by Narr et al. (2004), we began the hippocampal tracing in each hemisphere at the indentation of the hippocampal sulcus, or the middle point of the hippocampus in the coronal plane. We used the alveus of the hippocampus as the superior boundary. As the inferior boundary, we used the white matter of the parahippocampal gyrus. As the lateral boundary, we used the inferior temporal horn of the lateral ventricle. Finally, as the medial boundary, we used the ambient cistern. We continued hippocampal tracing posteriorly until the gray matter resembled an oval mass that was medial to the atrium of the lateral ventricles*. Bilateral hippocampi were traced three times, twice by a single graduate student tracer and once by an experienced postdoctoral tracer. The average measure intraclass coefficient (ICC) was 0.88 with a 95% confidence interval of 0.77–0.94 [*F*_(22, 66)_ = 8.03, *p* < 0.001] between all three tracings. Subsequently, since only two tracers were used a thresholded mask was created using only the voxels that were chosen in all three tracings. Volume data was extracted, using FSL utilities, from all masks and entered into SPSS.

The authors apologize for this error and state that this does not change the scientific conclusions of the article in any way.

## Conflict of interest statement

The authors declare that the research was conducted in the absence of any commercial or financial relationships that could be construed as a potential conflict of interest.
